# Estimating the Potential for Adaptation of Corals to Climate Warming

**DOI:** 10.1371/journal.pone.0009751

**Published:** 2010-03-18

**Authors:** Nikolaus B. M. Császár, Peter J. Ralph, Richard Frankham, Ray Berkelmans, Madeleine J. H. van Oppen

**Affiliations:** 1 Department of Environmental Sciences, Plant Functional Biology and Climate Change Cluster, University of Technology, Sydney, New South Wales, Australia; 2 Macquarie University, Sydney, New South Wales, Australia; 3 Australian Museum, Sydney, New South Wales, Australia; 4 Australian Institute of Marine Science, Townsville, Queensland, Australia; American Museum of Natural History, United States of America

## Abstract

The persistence of tropical coral reefs is threatened by rapidly increasing climate warming, causing a functional breakdown of the obligate symbiosis between corals and their algal photosymbionts (*Symbiodinium*) through a process known as coral bleaching. Yet the potential of the coral-algal symbiosis to genetically adapt in an evolutionary sense to warming oceans is unknown. Using a quantitative genetics approach, we estimated the proportion of the variance in thermal tolerance traits that has a genetic basis (i.e. heritability) as a proxy for their adaptive potential in the widespread Indo-Pacific reef-building coral *Acropora millepora*. We chose two physiologically different populations that associate respectively with one thermo-tolerant (*Symbiodinium* clade D) and one less tolerant symbiont type (*Symbiodinium* C2). In both symbiont types, pulse amplitude modulated (PAM) fluorometry and high performance liquid chromatography (HPLC) analysis revealed significant heritabilities for traits related to both photosynthesis and photoprotective pigment profile. However, quantitative real-time polymerase chain reaction (qRT-PCR) assays showed a lack of heritability in both coral host populations for their own expression of fundamental stress genes. Coral colony growth, contributed to by both symbiotic partners, displayed heritability. High heritabilities for functional key traits of algal symbionts, along with their short clonal generation time and high population sizes allow for their rapid thermal adaptation. However, the low overall heritability of coral host traits, along with the corals' long generation time, raise concern about the timely adaptation of the coral-algal symbiosis in the face of continued rapid climate warming.

## Introduction

Tropical coral reefs are among the most biodiverse ecosystems on the planet and of immense economic value to an estimated 500 million people worldwide [1]. The success of stony corals as carbonate depositing reef structures depends on their functional association with dinoflagellate symbionts of the algal genus *Symbiodinium*. This association is based on a closely linked nutrient cycling which promotes a near one-to-one ratio of algal photosynthesis and coral calcification rates [2]. Members (types or subclades) of four of the eight major *Symbiodinium* clades (clades A–D) are dominant among reef-building corals and largely influence holobiont (whole symbiosis) physiology [3].

Rapid deterioration of the world's tropical coral reefs is occurring due to multiple anthropogenic stressors. Of particular concern is the rising level of atmospheric greenhouse gases, especially CO_2_, resulting in global warming and ocean acidification [4], [5]. Warming of tropical seas has already pushed many coral species close to their upper thermal limit [6]. Sea surface temperatures (SSTs) that exceed summer maxima by 1–2°C over a few weeks usually cause severe bleaching of corals due to the loss of algal symbionts and/or algal pigments [7]. Currently, a third of all reef-building coral species is threatened by an increased risk of extinction [8]. Some of the most temperature-sensitive and bleaching-susceptive coral species belong to the family Acroporidae, which are among the most important contributors to Indo-Pacific coral reefs in terms of species richness and biomass [9], [10]. Of these, an estimated 50% of species are at particularly high risk of extinction [8].

Sea surface temperatures are predicted to further rise by 2–3°C over the 21^st^ century [5]. Therefore, it has been suggested that, unless there is substantial thermal adaptation of coral hosts and/or their associated symbionts [11], [12], large scale bleaching and mortality events could occur annually on the world's coral reefs by 2050 [13].

Both symbiotic partners can mitigate the damaging effects of thermal stress by employing protective mechanisms such as increased expression of antioxidant enzymes, protective pigments and heat shock proteins [14]. For example, the coral's bleaching response is thought to be triggered by its symbionts, whose photosynthetic apparatus becomes increasingly damaged under thermal stress both in photosystem II (PSII) [15], and in the dark reactions of photosynthesis [16]. This process is called photoinhibition [17], and thereby accumulating reactive oxygen species (ROS) initiate the symbionts' antioxidant response. The symbionts' antioxidant response neutralises these harmful ROS via enzymes such as superoxide dismutases (SODs) [14]. Also, non-photochemical quenching (NPQ) of light energy, either through regulated (ΦNPQ) or unregulated (ΦNO) thermal energy dissipation [18], allows for the diversion of excess excitation energy away from the symbiont's PSII reaction centre. A key NPQ mechanism (i.e. ΦNPQ) is the reversible conversion of the xanthophyll pigment diadinoxanthin (DD) into the photoprotective diatoxanthin (DT) [19]. This xanthophyll cycle reduces photosynthetic efficiency by dissipating excess excitation energy in the form of heat within the PSII antenna; thus suppressing oxidative damage to the photosynthetic apparatus [20]. Unregulated non-photochemical quenching (ΦNO) represents a passive energy dissipation mechanism in the form of heat mainly due to closed PSII reaction centres [21]. If the symbionts' photoprotective mechanisms are overwhelmed ROS inevitably leak through to coral host cells. Symbiont-derived ROS, as well as ROS produced by damaged coral mitochondria initiate the coral's up-regulation of antioxidants (e.g. manganese SOD, MnSOD) and/or heat shock proteins (e.g. Hsp70) to prevent and/or to repair cellular damage [14]. Although the signalling events that actually lead to bleaching are yet not fully understood, there is strong evidence that ROS levels exceeding the coral's stress defence result in bleaching [14], [22].

Thermal- and bleaching sensitivities of corals often correlate with physiological properties of the accommodated symbiont type. However, bleaching responses can vary even between congeneric [23] or conspecific [24] corals hosting the same symbiont type. This suggests a role for the coral host in influencing the bleaching response, but the relative contribution of the host versus the symbiont to the holobiont's overall thermal tolerance has not been examined [25]. Further, it is unknown whether this observed phenotypic variation (*V_P_*) in thermal tolerance is mainly due to environmental (*V_E_*) or genetic factors (*V_G_*), information which is crucial to predicting the potential for adaptation through natural selection. Genetic variation (*V_G_*) in functional traits is the currency for natural selection that allows populations to adapt to environmental changes [26]. Thus, it is the genetic basis underlying phenotypic stress mitigation mechanisms that determines the adaptive potential for thermal tolerance in the coral-algal symbiosis.

In long-lived, clonal species such as corals the adaptive potential is best approximated by the clonal or broad-sense heritability (*H^2^ = V_G_/V_P_*); i.e. the proportion of phenotypic variation that is due to genetic factors [27]. We estimated *H^2^* of a suite of vital phenotypic traits related to thermal tolerance in two distinct populations of the widespread Pacific stony coral *Acropora millepora* under bleaching conditions (32°C). Table 1 lists the eleven functional traits investigated and their main biological roles. We designate traits as those of the algal symbiont, the coral host and the holobiont, according to where the major control of the trait resides. However, it is unlikely that any ‘symbiont’ or ‘host’ trait is entirely independent of the other. For example, the symbionts live within coral host cells and the host therefore has direct influence over their physical and chemical environment. Nevertheless, certain traits such as photosynthetic properties and coral stress gene expression are determined to a larger extent by the symbiont or the host, respectively [sensu 25]. Coral calcification rates are tightly coupled to photosynthesis [28], hence we assume here that coral growth is contributed to by both symbiotic partners, and we will refer to it as a holobiont trait.

**Table 1:** Thermal tolerance traits investigated.

	Trait	
Symbiont	Photochemistry	Fv/Fm - maximum quantum yield of photosystem II (PSII)
		ΦPSII - effective quantum yield of PSII
		ΦNPQ - regulated non-photochemical quenching of excitation energy
		ΦNO - unregulated non-photochemical quenching of excitation energy
	Pigment profile	Xanthophyll cycling, expressed as molar ratios of DT/(DD+DT) - provides a measure of photosynthetic thermal energy dissipation through the reversible conversion of diadinoxanthin (DD) into the thermo-protective diatoxanthin (DT)
		Relative pool sizes of photoprotective xanthophyll (XP; i.e. DD and DT) to total light-harvesting pigments (LH; i.e. *chlorophyll* a, *chlorophyll* c2, peridinin, DD, and DT), expressed as molar ratios of XP/(LH+XP)
Coral host	Gene (mRNA) expression	Ferritin - an iron-binding protein which maintains cellular redox balance by minimising Fe^2+^ availability for generating harmful oxygen radicals
		Hsp70 - heat shock protein 70, prevents protein denaturation during thermal stress
		MnSOD - manganese superoxide dismutase, a mitochondrial antioxidant enzyme
		Zn^2+^-met - zinc-metalloprotease, might be involved in the dysfunction of coral cell-adhesion proteins during bleaching via a remodelling of surface receptors in the extra-cellular matrix
Holobiont	Growth rates	Defined as percent increase in buoyant weight of combined coral tissue and skeleton

Traits reflecting the function of the algal symbiont included four traits related to photochemistry, and two traits related to antenna pigment profile. Coral host traits comprised mRNA gene expression patterns of four essential key genes involved in the oxidative stress response (Csaszar *et al.* 2009). Coral colony growth was measured as a holobiont (whole symbiosis) trait.

Both *A. millepora* populations investigated inhabit different thermal environments in the central Great Barrier Reef (GBR), Australia. One is from a warmer site (Magnetic Island; mean summer SST ∼29.5°C) and associates with the thermo-tolerant *Symbiodinium* clade D, while the other, from a cooler site (Orpheus Island; mean summer SST ∼28.7°C), associates with the intermediately tolerant *Symbiodinium* type C2. Both populations were investigated in separate, highly controlled ‘common garden’ experiments, with the Magnetic Island (MI) population being examined in winter (August/September 2006), and the Orpheus Island (OI) population in autumn (April/May 2008). Thus, the evolutionary potential of the two coral-algal populations cannot be directly compared. Instead, we investigate the degree of genetic determination of phenotypes in the two populations at their respective time of assessment, under the particular environmental conditions experienced. Our results provide insights into the relative evolutionary potential of the coral host, its associated algal symbionts, and the holobiont to genetically adapt to warming oceans.

## Results

Both coral-algal populations showed a similar trend for their capacity to adapt to warming oceans. Overall, algal symbionts belonging to *Symbiodinium* clade D (Magnetic Island) and type C2 (Orpheus Island) displayed a substantially higher potential than their coral host *Acropora millepora* for genetic adaptation to bleaching conditions (32°C) (Table 2). Either symbiont type showed significant heritability in five of six functional key traits, with the average estimate being higher in D (*H^2^* = 0.46±0.03) compared to C2 symbionts (*H^2^* = 0.35±0.05). This is in stark contrast to the coral host, which, in both populations, lacked heritability for the majority of traits investigated; i.e. with only one of four traits showing significant heritability (Table 2).

**Table 2:** Heritability of thermal tolerance traits.

		MI (clade D)		OI (type C2 )	
	Trait	Heritability	SE	Heritability	SE
Symbiont	Fv/Fm	0.50*	0.084	0.30	0.111
	ΦPSII	0.43*	0.092	0.34*	0.112
	ΦNPQ	0.31	0.101	0.23*	0.117
	ΦNO	0.36*	0.098	0.34*	0.109
	DT/(DD+DT)	0.54*	0.079	0.53*	0.089
	XP/(LH+XP)	0.48*	0.086	0.30*	0.113
Coral host	Ferritin	0.04	0.112	0.06	0.124
	Hsp70	0.14	0.110	0.15	0.121
	MnSOD	0.18*	0.109	0.06	0.124
	Zn^2+^-met	0.16	0.110	0.34*	0.110
Holobiont	Growth rates	0.59*	0.073	0.19*	0.115

Thermal tolerance traits in the Magnetic Island (MI), and the Orpheus Island (OI) population of *Acropora millepora* and their respective symbiont populations (*Symbiodinium* clade D and type C2) under bleaching conditions (32°C). Broad-sense heritabilities and their standard errors (SE) are shown. Significant heritabilities are indicated bold with asterisks (*), and values usually vary between zero and one (0 = all variation due to environmental variation; 1 = all variation due to genetic factors). Phenotypic variation in natural populations is typically caused by both genetic and environmental factors. Genetic variation is the working-substrate for natural selection that facilitates adaptation.

### Symbiont traits

Traits reflecting the function of the symbiont *in hospite* (within the host) included four parameters related to photosystem II (PSII) photochemistry, and two traits concerning PSII antenna pigment profile.

In the MI symbiont population, maximum quantum yield of PSII (Fv/Fm) showed significant heritability (*H^2^* = 0.50, P<0.001) at bleaching temperatures (32°C), suggesting a high potential for thermo-tolerant clade D symbionts to adaptively respond to selection for Fv/Fm under thermal stress (Table 2). The intermediately tolerant OI symbiont population did not display significant heritability for this stress parameter. Therefore, it seems that genetic factors (*V_G_*) do not contribute significantly to phenotypic Fv/Fm performance in C2 symbionts under bleaching conditions.

However, C2 symbionts showed significant heritabilities in all three complementary light-adapted energy pathways, thus allowing in the OI population for adaptive changes to the effective quantum yield of PSII (ΦPSII; *H^2^* = 0.34, P = 0.010), to PSII down-regulation (ΦNPQ; *H^2^* = 0.23, P = 0.030), and to unregulated dissipation of PSII excitation energy (ΦNO; *H^2^* = 0.34, P = 0.012) (Table 2). While both ΦPSII and ΦNO also showed significant heritabilities in D symbionts (*H^2^* = 0.43, P = 0.010 and *H^2^* = 0.36, P = 0.020, respectively), there was no significant genetic variation for ΦNPQ, suggesting there is no adaptive potential for this latter parameter in the MI population.

The ability to change PSII pigment composition and, thus, to increase photoprotection under thermal stress showed an evolutionary potential in either symbiont type. In D symbionts, heritability was high for xanthophyll cycling (i.e. molar DT/(DD+DT) ratios; *H^2^* = 0.54, P<0.001), and the ability to change relative pool sizes of xanthophyll to total light-harvesting pigments (i.e. molar XP/(LH+XP) ratios; *H^2^* = 0.48, P<0.001). Similarly, in C2 symbionts, the regulation of both DT/(DD+DT) and XP/(LH+XP) ratios yielded significant heritability estimates (*H^2^* = 0.53, P = 0.002 and *H^2^* = 0.30, P = 0.003, respectively), thus suggesting an overall high adaptive potential for photoprotective measures in the two different symbiont types.

### Coral host traits

Of all the four major antioxidant and stress genes investigated in the MI coral host population, only the expression of the antioxidant enzyme MnSOD showed a significant, although moderately high heritability under thermal stress (*H^2^* = 0.18, P = 0.031) (Table 2). Likewise, in the OI host population, heritabilities for gene expression patterns were not significant except for one gene, the metalloprotease Zn^2+^-met (*H^2^* = 0.34, P = 0.003). Therefore, in either of the two *A. millepora* populations, the coral host itself has only a low potential for adaptive changes of these cellular thermal-stress defence mechanisms.

### Holobiont trait

Significant, although variably high heritabilities for coral growth at bleaching temperatures were found both in the MI (*H^2^* = 0.59, P<0.001) and in the OI holobiont population (*H^2^* = 0.19, P = 0.033), indicating the presence of genetic variation and, thus, an evolutionary potential for this trait (Table 2).

## Discussion

The major finding of our study is that coral-associated symbionts, which are generally considered to initiate the coral bleaching response [15], [16] possess a substantially higher potential for thermal-stress adaptation than their coral animal host. This appears to put *A. millepora* into a half-full glass situation, in which the adaptive potential to climate warming is largely determined by the algal partner of the symbiosis. In this respect, a recent quantitative genetic modelling study [29] showed that, with genetic variation (*V_G_*) in symbiont thermal tolerance, corals might be able to persist into the next century. However, only if current greenhouse gas emissions are reduced [29] and, the authors further caution that the multiple anthropogenic stressors on corals other than elevated temperature are not accounted for in their model. The present study established that coral associated symbionts show heritable variation in thermal tolerance traits. Thus, the first of these two model assumptions for the potential timely adaptation of corals to climate warming are clearly met [sensu 29].

### Symbiont traits

Maximum photosynthetic quantum yield (Fv/Fm), yielding a significant heritability in clade D symbionts only, is one of the most commonly used stress parameters in coral biology as it indicates the proportion of functional PSII reaction centres and, thus, levels of photoinhibition considered to trigger coral bleaching [15]. D symbionts also displayed heritability for the effective quantum yield of photosynthesis under illumination (ΦPSII), and for passive dissipation of excess excitation energy (ΦNO). The only trait that did not yield a significant heritability estimate in D symbionts was ΦNPQ. Non-photochemical quenching of light energy (ΦNPQ) reflects a measure to protect algal photosystems from over-excitation whereas increased levels of ΦNO, at the cost of lowered ΦPSII and/or ΦNPQ, are indicative of photoinhibition [21]. Depending on whether natural selection favours increases or decreases in ΦPSII and ΦNO, corresponding decreases or increases in ΦNPQ might indirectly accompany changes in these three complementary traits. Such an indirect effect on the distribution of a particular trait in a population is commonly observed when selection acts upon a correlated trait [30]. Ideally, if selection favours a down-regulation of ΦNO (and an unchanged ΦPSII), an evolutionary increase in ΦNPQ might occur.

Regardless of such potential evolutionary trajectories in D symbionts, by displaying heritable genetic variation for xanthophyll cycling (i.e. DT/(DD+DT) ratios), their adaptive potential for photoprotection is evident with respect to changes to PSII antenna pigment composition. Both the xanthophyll cycle and ΦNPQ might be considered as correlated traits, with the latter being controlled by the acidification of the chloroplast thylakoid lumen as a result of photosynthetic electron transport, which in turn initiates the xanthophyll cycle [20]. Therefore, while D symbionts do not possess the potential to further evolve the ‘trigger’ to operate the xanthophyll cycle (i.e. ΦNPQ) at higher efficiency under bleaching conditions, they possess the potential for adaptive changes to the efficiency of the actual xanthophyll cycling mechanism itself. Moreover, the ability of D symbionts to increase their photoprotective xanthophyll pool size (XP) relative to that of total light harvesting pigments (LH; including XP) under bleaching conditions also showed an evolutionary potential. Increased XP pool sizes are a measure assumed to aid in physically shielding the cell, rather than absorbing light energy [31]. Xanthophylls might even have additional photoprotective functions beyond non-photochemical quenching and cell shading, such as stabilisation of the thylakoid membrane [32] and antioxidant activity through quenching of ROS [33]. Evolutionary changes to the symbionts' photosynthetic/photoprotective pigment composition can therefore be assumed to influence thermal tolerance.

As with clade D symbionts, type C2 symbionts from the OI population showed heritability in almost all the investigated thermal tolerance traits. The one trait that did not yield a significant estimate in C2 symbionts was Fv/Fm. However, all three light-adapted fluorescence parameters showed heritability, thus allowing for adaptive changes in energy utilisation of photosynthesis (ΦPSII), for all non-photochemical energy quenching measures that are not photoprotective (ΦNO), and for initiating the xanthophyll cycle via regulated energy dissipation (ΦNPQ). C2 symbionts also displayed heritability for the xanthophyll cycling mechanism as well as for the ability to change the ratio of photoprotective to total light-harvesting pigments. Therefore, with a significant potential for adaptation in most of the traits investigated, the basis for evolutionary changes to major components of both photosynthesis and photoprotection in response to elevated seawater temperatures is evident in both symbiont types. Yet it remains to be seen whether potential symbiont adaptations might suffice for maintaining a functional holobiont under continued rapid climate warming [sensu Baskett *et al.* 2009].

### Coral host traits

Both host populations of *A. millepora* displayed low overall levels of quantitative genetic variation for the expression of almost all four stress and antioxidant genes investigated. Manganese SOD, which usually constitutes the first line of cellular defence against ROS [34], was the only gene to show a low, although significant heritability for its expression in the MI coral host population. Under severe stress, the primary antioxidant defence might be overwhelmed and, therefore, secondary mechanisms are mobilised to counter oxidative damage by repairing and replacing dysfunctional molecules [14]. This secondary defence response in the form of the synthesis of Hsp70, the most conserved heat shock protein [35], did not yield a significant heritability estimate in either coral host population. Neither did the expression pattern of the main cellular iron storage protein Ferritin, which usually binds free Fe^2+^ and thus prevents further ROS production and accumulation [36]. In the OI host population, the only expression pattern that showed heritability was that of the matrix metalloprotease Zn^2+^-met. Of all the investigated genes, the physiological roles of these metalloproteases are the least understood [but see 37]. Due to their proteolytic activity in the extra-cellular matrix (ECM), zinc-metalloproteases might be involved in the coral host's control over population densities *in hospite* under thermal-stress conditions. For example, through functional changes to coral cell-adhesion proteins [38] and hence a potential involvement in the expulsion of symbiont containing cells during bleaching. Alternatively, zinc-metalloproteases might be involved in the disruption of the signal transduction required for the communication between host and symbiont that usually maintains the symbiosis [39].

It is important to note, that the four genes investigated represent but a small subset of the coral's entire transcriptome [see 37], and the relationship between gene expression and physiological processes is complex [40]. Yet Hsp70 and MnSOD are among the most fundamental and frequently used coral stress biomarkers [14]. Additionally, even though minor evolutionary changes in gene expression are likely to be biologically significant [40] the fact remains that the majority of the four genes investigated did not show a significant adaptive potential.

No comparable studies exist for heritabilities of gene expression patterns in corals. For other organisms, the study by Cammack *et al*. [41] is probably comparable; the authors also applied a qRT-PCR assay and estimated a heritability of ∼0.30 for the testicular expression of a heat shock protein (Hspcb) in male mice. In the fruit fly *Drosophila melanogaster*, a study that used an enzyme linked immuno-absorbent assay (ELISA), found significant broad-sense heritabilities for protein levels of Hsp70 ranging from 0.25–0.49 in adult and third-instar larval stages, respectively [42]. For haemoglobin levels of antioxidant SODs (i.e. CuZnSOD) in sheep, narrow-sense heritabilities (i.e. the proportion of *V_P_* that is due only to additive genetic variation, *V_A_*) of ∼0.19 have been found [43]. Unfortunately, none of these studies estimated heritability for the expression of both antioxidant enzymes and heat shock proteins simultaneously in the same organism. It is therefore unknown whether it is a common pattern for organisms to show heritability for just a few genes involved in the oxidative stress response, as observed for coral gene expression in the present study.

In other organisms such as yeast, maize, mice, and humans, gene expression patterns generally tend to show higher levels of heritability (∼50% of all expressed loci at any given time) [44]–[46]. Recent adaptation to increasing SSTs might explain the overall low adaptive capacity in the two coral host populations investigated. On the GBR, recurrent and large-scale coral bleaching events have been recorded on reef-sites including both MI and OI over the past three decades [47]–[49]. As a result, beneficial alleles within each coral population might already be more or less fixed at the relevant functional gene loci. This is likely to lead to the observed low levels of heritability for thermal tolerance traits in the present study, and was also the likely cause in two rainforest species of *Drosophila*, where the most resistant populations showed essentially no genetic variation in response to climatic stress [50] even after intense selection for over 30 generations [51].

Whenever genetic variation for functional traits is determined solely by a mutational input of beneficial alleles (as opposed to standing levels of genetic variation), as it is the case for the majority of the investigated host traits in *A. millepora*, the rate of environmental change to which a population might be able to adapt to might be as low as one percent per generation [52], [53]. Also, this potential evolutionary response is provided only if (effective) population sizes are large enough. Assuming that coral population sizes are large enough, and that levels of gene flow among populations allow local adaptation [54], long-lived organisms such as reef-building corals are nevertheless very likely to encounter environmental changes greater than one percent during their lifetime, even more so in these times of rapid climate warming.

### Holobiont trait

Both holobiont populations showed heritability for coral weight gain at 32°C, suggesting an adaptive potential for colony growth rates under thermal stress. Heritability in this trait is crucial as continuous coral growth maintains the complex three-dimensional reef structure, which represents a delicate balance between continuous coral calcification rates and destructive physical and biological forces [55]. Despite the heritability found for this trait, however, coral growth rates in the long term could ultimately be limited by environmental constraints such as the seawater's calcium carbonate saturation state. Marine carbonate saturation might be lowered beyond the critical threshold for coral calcification in the latter half of the 21^st^ century due to ocean acidification [55], [56]. The corals' immediate response to a lower calcium carbonate saturation state is a reduction in the linear extension rate and density of the skeleton, resulting in more fragile colonies that are more vulnerable to erosion [56], [57]. Yet corals might maintain both skeletal growth and density by investing greater energy in calcification [56], although this strategy likely requires the diversion of resources from other imperative biological functions such as reproductive output [57]. Nevertheless, at present, the genetic variation (*H^2^*) that has been found to underlie coral (holobiont) growth rates will certainly allow adaptive changes to increasing temperatures.

### Implications

For traits with significant heritability, thermal adaptations between both sexual and asexual generations of the animal host and its algal symbiont will occur at different temporal scales due to the fundamentally different life histories of the two partners. Short clonal turnover times of symbionts (≤2 weeks *in hospite*) [58] allow for rapid genetic adaptation, although the symbionts' mainly asexual reproduction *in hospite* restricts the genetic response (i.e. the evolutionary change) to that of the most thermo-tolerant clone available within the existing population. An increased probability for the occurrence of somatic mutations due to the symbionts' large population sizes (≥10^6^ cells cm^−2^ coral tissue) [58] might increase a given genetic response to selection modestly. There is growing evidence that *Symbiodinium* can reproduce sexually in the free-living stage [reviewed in ref. 59], which is likely to increase genetic variation in free-living populations. Also, symbionts are acquired anew horizontally from the environment during early life stages in the majority of reef-building corals (∼85%; including *A. millepora*) [60]. Therefore, while adaptive symbiont improvements cannot be directly passed on to the next sexual coral generation by the parental colonies, the opportunity is given in each successive generation to establish symbiosis with locally better adapted *Symbiodinium* genotypes [61], [62].

Rates for evolutionary changes in holobiont and host traits, on the other hand, will inevitably lag behind those for symbiont adaptation due to the longer generation time of the coral animal (∼4 yrs for acroporids, >20 yrs for the majority of corals) [63]. *A. millepora* reproduces mainly sexually on the GBR. While this in itself might eventually (via gene segregation and recombination) generate genotypes superior to solely clonal offspring, it delays the establishment of superior genotypes within existing coral populations over successive host generations (as opposed to faster clonal reproduction). Therefore, and due to the mostly insignificant levels of standing genetic variation for combating cellular stress, overall chances for sufficient and rapid adaptive responses in the coral host seem to be low. Hence, there is warranted doubt as to whether thermal adaptation of coral hosts might occur in time to match predicted rates of rapid climate warming. The alarmingly low overall evolutionary potential of the coral highlights the urgency for drastically reducing anthropogenic pressures on corals and green-house gas emissions if coral reefs are to persist.

## Materials and Methods

### Collection of corals, genotyping of symbionts and coral hosts

Four pairs of adjacing coral branches (i.e. nubbins of 3–4 cm length) from each of twenty colonies of *Acropora millepora* from a mid-shelf reef location from Magnetic Island (MI; Nelly Bay - 19°09.74' S/146°51.33' E), and Orpheus Island (OI; Cattle Bay - 18°34.348 S/146°29.029 E) were collected from 3–5 m depth using SCUBA, and transferred to the Australian Institute of Marine Science (AIMS, Townsville) for subsequent comparative testing (see next section).

Additional nubbins from each of the twenty colonies were removed for genotyping. Prior to estimating broad-sense heritabilities (*H^2^*) for phenotypic traits within populations, genetic heterogeneity among the coral colonies investigated must be verified since trait variation needs to be measured within and between genetically unique individuals (genotypes) [27]. Additionally, a single, dominant symbiont sub-clade across all genetically heterogeneous coral colonies, as opposed to a consortium of symbiont types varying in both quality and quantity, allows for the deduction of phenotypic trait data within each population to a single cause, thereby minimizing additional variance components that cannot be accounted for otherwise.

Identification of *in hospite* symbionts at the sub-cladal level was accomplished by analysing sequences of the nuclear ribosomal DNA internal transcribed spacer 1 (rDNA ITS1) as described [64] using single-strand conformational polymorphism (SSCP) [65]. However, the low resolution of ITS sequences prevents identification of individual genotypes within zooxanthellae sub-clades, thus assumptions for heritability estimates are made (described later). *Acropora millepora* colonies from MI harboured exclusively type D *Symbiodinium* (GenBank Accession EU024793), whereas coral colonies from Orpheus Island contained only type C2 *Symbiodinium* (GenBank Accession AF380552) [sensu 64]. Microsatellite genotyping [66] confirmed a unique multi-locus genotype and thus genetic heterogeneity among the twenty individual coral colonies in either host population.

### Heat-stress experiment

In both heat-stress experiments, four clonal nubbins from each of the 20 coral colonies were arranged in indoor tanks in a randomized block design, resulting in n = 80 coral nubbins for each of the two temperature treatments (27°, and 32°C). Lights were mounted above each tank and provided an average underwater light intensity of 130 µmol photons m^−2^ s^−1^ on a 10∶14 hrs light∶dark cycle (10×400 W metal halide lamps, BLV, Germany). The lights were UV-filtered in order to avoid radiation induced bleaching [67], and to measure exclusively the effects of thermal stress. Tanks were supplied with fresh filtered (1 micron) seawater, which was held at 27°C (±0.1°C) via computer control [68] using a flow-through system at a rate of 1.2 litres min^−1^. Coral nubbins were given an indoor acclimation period of two weeks prior to exposure to 32°. The tanks were slowly ramped up to this target temperature (±0.02–0.05°C) via computer control [68] over a period of 6 days, and the total duration of the heat-stress experiment (including the slow ramp) was 15 days.

All clonal replicates (n = 4 each) from two out of the initial 20 *A. millepora* colonies from OI suffered mortality throughout the heat-stress experiment, thus restricting the subsequent heritability analysis for the OI population to 18 coral genotypes.

### Photosynthesis parameters, coral growth

Light energy that hits photosystem II (PSII) can be used to either drive photochemistry or, if in excess, can be re-emitted as fluorescence light or dissipated as heat. Non-invasive pulse amplitude modulated (PAM) fluorometry allows for measuring the corresponding three complementary energy pathways and gives information about the actual efficiency of PSII (ΦPSII), regulated (ΦNPQ) and unregulated (ΦNO) energy dissipation in the light-adapted state [18], as well as the maximum dark-adapted quantum yield of PSII fluorescence (Fv/Fm).

Dark-adapted maximum quantum yield (Fv/Fm) of photosynthesis was measured every other day at the same time in the morning (08:00–10:00 am) using an Imaging-PAM (Walz, Germany) [69]. The three light-adapted parameters add up to unity (ΦPSII + ΦNPQ + ΦNO = 1) [18], and were measured accordingly on the same days in the afternoon (after exposure to light for 3 hrs). The percent change in each of those parameters at the end of the experiments was assessed relative to their respective initial value. Imaging-PAM settings were: measuring light intensity = 2, saturation pulse intensity = 5, gain = 1, damping = 2.

Growth rates of corals were treated as a holobiont trait using the buoyant weighing technique [70]. The final weight of each nubbin after the experiment was compared to its initial weight (after the temperature ramp when the target temperature was reached), and coral growth rates were defined as the % overall weight increase (combined coral tissue biomass and skeleton weight) over time.

### Pigment ratios, coral gene expression

Coral samples were bleached after the temperature experiment (15 d), and compared to a series of control samples that were removed before the temperature ramp at T_0_ (27°C). Pairs of neighbouring nubbins were chosen for the post-experimental comparison of T_0_ and bleached samples based on the assumption that neighbouring nubbins from the same colony do not only share an identical genotype, but were also exposed to a similar micro-climate in the field. Thus, due to their shared similar developmental history, immediately neighbouring nubbins within the same coral colony should resemble each other physiologically more than more distant parts of the same colony. Both T_0_ (control) and bleached coral nubbins were ground in liquid nitrogen, and the crude powder was stored at −80°C.

After methanol (MeOH) extraction, symbiont xanthophyll pigments were separated with an HPLC (Waters 600, Waters Corp., Massachusetts, USA) linked to a photodiode array detector (Waters PDA 996; Waters Corp., Massachusetts, USA) using a 3 µm analytical reverse phase column (Phenomenex Gemini C18, Phenomenex Inc., California, USA). The HPLC solvent consisted of solvent A (70∶30 v/v MeOH∶28 mM aqueous tetrabutyl ammonium acetate – TBAA, pH6.5) and solvent B (50∶50 v/v MeOH∶acetone) with a flow rate of 1ml min^−1^, and 20 µl injection volume. Eluted peaks were monitored at a wavelength of 440 nm, and peak identities were determined by comparing retention times with pigment standards (Sigma-Aldrich). Peak area was integrated using EMPOWER software (Waters Corp., Massachusetts, USA), and absorbance ratios at 440 nm adjusted to molar ratios.

For coral gene expression, total mRNA was extracted using Dynabeads® Oligo(dT)_25_ (Invitrogen™) as described [37]. Complementary DNA (cDNA) was constructed from normalized mRNA quantities using SuperScript™ III First-Strand Synthesis SuperMix (Invitrogen™) according to the manufacturer's protocol. Specific forward and reverse primers for both the four target genes (i.e. Ferritin, Hsp70, MnSOD, and Zn^2+^-met; GenBank accession numbers DY585921, DY581262, DY584495, and DY581364, respectively) and two internal control genes (Ctg 1913 (GenBank DY585358) and RiboL9 (DY586572) in the MI population; RiboL9 and GAPDH (DY578497) in the OI population) were designed (90–110 bp amplicon length, ∼55% GC content, 65°C primer Tm) and commercially obtained from Sigma-Aldrich™. Each cDNA sample was used in triplicate 20 µL qRT-PCR reactions with 1 µL (4 µM) primers, 6 µL UV-sterilized Milli-Q® water, and 10 µL SYBR® GreenER™ qPCR SuperMix Universal on a Rotor-Gene™ RG-3000A (Corbett Research™). Threshold cycle differences (ΔCt) were obtained by manually positioning the threshold immediately above the background noise in the exponential amplification phase. The efficiency corrected relative expression of each of the four target genes in relation to the geometric mean of the two housekeeping genes was calculated using *geNorm* software [71].

### Heritability

We calculated *H^2^* by partitioning phenotypic variance (*V_P_*) into genetic (*V_G_*) and environmental variance (*V_E_*) components based on analyses of variance (ANOVA) involving among and within coral clone variation. Variation within clones provides an estimate of *V_E_*, while that among colonies is due to *V_G_+V_E_*. Consequently, we estimated the genetic variation among colonies by subtracting the environmental variance from the total phenotypic variance (*V_G_ = V_P_-V_E_*), and calculated heritability as *V_G_/V_P_* [27]. Tables S1, Table S2, Table S3, Table S4 and Table S5 show the ANOVA tables for the heritability analysis.

An acclimation period of genotypes to a common environment prior to measuring phenotypic variables minimises additional environmental variance components such as carry-over effects from the field and maternal effects [27]. The corals in the present study have been acclimatised to a common outdoor environment for 2 weeks prior to another 2 weeks of acclimation to highly controlled indoor conditions. However, if some environmental effects, despite this long acclimation period, have been carried over into the laboratory environment heritabilities might be overestimated. Also, heritability in symbiont traits may be biased due to uncertainties about genotypic variation within zooxanthellae sub-clades. Thus, the assumption is made here that juvenile corals in either population have been invaded during their ontogeny by more than one symbiont genotype of respectively *Symbiodinium* clade D and type C2. There may be little bias in heritability estimates if those symbionts are approximately equally dispersed across the colony surface landscape such that every individual coral nubbin harbours a similar consortium of symbiont genotypes. However, if there is only one distinct symbiont genotype per coral colony then *H^2^* will be biased upwards while the presence of multiple genotypes that are not uniformly transmitted throughout each coral colony (i.e. such that individual nubbins harbour distinct symbiont genotypes) will lead to *H^2^* estimates that are biased downwards.

## Supporting Information

Table S1 ANOVA table for maximum dark-adapted fluorescence yield (Fv/Fm) and coral (holobiont) growth.
(0.05 MB DOC)

Table S2 ANOVA table for light-adapted fluorescence yields (ΦPSII, ΦNPQ, and ΦNO).
(0.07 MB DOC)

Table S3 ANOVA table for symbiont pigment ratios (DT/(DD+DT) and XP/(LH+XP)).
(0.05 MB DOC)

Table S4 ANOVA table for coral host gene expression (Magnetic Island).
(0.05 MB DOC)

Table S5 ANOVA table for coral host gene expression (Orpheus Island).
(0.05 MB DOC)
