# Supplementary material for: Estimating the Potential for Adaptation of Corals to Climate Warming
Source: PLoS One. 2010 Mar 18;5(3):e9751. doi: 10.1371/journal.pone.0009751 (PMC2841186; doi:10.1371/journal.pone.0009751)
Supplement: Table S1 — ANOVA table for maximum dark-adapted fluorescence yield (Fv/Fm) and coral (holobiont) growth. (0.05 MB DOC) [file pone.0009751.s001.doc]

**Table S1**

|  |  | | **Orpheus Isl. (*Symb*. C2)** | | | | | **Magnetic Isl. (*Symb*. D)** | | | |
| --- | --- | --- | --- | --- | --- | --- | --- | --- | --- | --- | --- |
|  |  | | **SS** | **df** | **MS** | | **p** | **SS** | **df** | **MS** | **p** |
| **Fv/Fm** | Variance (colonies) | between | 257.36241 | 17 | 15.13896 | | 0.292 | 1278.878 | 19 | 67.30939 | **<0.001** |
|  |  | within | 612.51125 | 49 | 12.50023 | |  | 1022.786 | 60 | 17.04643 |  |
|  | Variance (tanks) | between | 355.24933 | 3 | 118.41644 | | <0.001 | 261.757 | 3 | 87.25244 | 0.026 |
|  |  | within | 514.62433 | 63 | 8.16864 | |  | 2039.907 | 76 | 26.84088 |  |
|  | Adjusted error terms  Total phenotypic variance (V_P_) | | 257.26200 | 46 | 5.59265 | |  | 761.029 | 57 | 13.35139 |  |
|  |  |  | 7.979239 | | | | | 26.84079 | | | |
| **Coral growth** | Variance (colonies) | between | 8.10180 | 17 | 0.47658 | **0.033** | | 80.580 | 19 | 4.24106 | **<0.001** |
|  |  | within | 12.65646 | 52 | 0.24339 |  | | 43.610 | 60 | 0.72683 |  |
|  | Variance (tanks) | between | 0.59144 | 6 | 0.19715 | 0.589 | | 7.687 | 3 | 2.56221 | 0.180 |
|  |  | within | 20.16681 | 66 | 0.30556 |  | | 116.503 | 76 | 1.53293 |  |
|  | Adjusted error terms  Total phenotypic variance (V_P_) | | 12.06502 | 49 | 0.24622 |  | | 35.923 | 57 | 0.630228 |  |
|  |  |  | 0.303814 | | | | | 1.532921 | | | |
