# Supplementary material for: Estimating the Potential for Adaptation of Corals to Climate Warming
Source: PLoS One. 2010 Mar 18;5(3):e9751. doi: 10.1371/journal.pone.0009751 (PMC2841186; doi:10.1371/journal.pone.0009751)
Supplement: Table S2 — ANOVA table for light-adapted fluorescence yields (ΦPSII, ΦNPQ, and ΦNO). (0.07 MB DOC) [file pone.0009751.s002.doc]

**Table S2**

|  |  | | **Orpheus Isl. (*Symb*. C2)** | | | | **Magnetic Isl. (*Symb*. D)** | | | |
| --- | --- | --- | --- | --- | --- | --- | --- | --- | --- | --- |
|  |  | | **SS** | **df** | **MS** | **p** | **SS** | **df** | **MS** | **p** |
| **ΦPSII** | Variance (colonies) | between | 404.87398 | 17 | 23.81612 | **0.010** | 282.209 | 19 | 14.853 | **0.010** |
|  |  | within | 496.29791 | 49 | 10.12853 |  | 392.882 | 59 | 6.659 |  |
|  | Variance (tanks) | between | 139.50024 | 3 | 46.50008 | 0.014 | 180.135 | 3 | 60.045 | <0.001 |
|  |  | within | 761.67165 | 63 | 12.09003 |  | 494.956 | 75 | 6.599 |  |
|  | Adjusted error terms  Total phenotypic variance (*V_P_*) | | 356.79767 | 46 | 7.75647 |  | 212.747 | 56 | 3.799 |  |
|  |  |  | 11.771383 | | | | 6.56254 | | | |
| **ΦNPQ** | Variance (colonies) | between | 604.59138 | 17 | 35.56419 | **0.030** | 1193.564 | 19 | 62.819 | 0.112 |
|  |  | within | 831.71817 | 47 | 17.69613 |  | 2483.882 | 60 | 41.398 |  |
|  | Variance (tanks) | between | 116.28843 | 3 | 38.76281 | 0.158 | 1407.756 | 3 | 469.252 | <0.001 |
|  |  | within | 1320.02112 | 61 | 21.63969 |  | 2269.691 | 76 | 29.864 |  |
|  | Adjusted error terms  Total phenotypic variance (*V_P_*) | | 715.42974 | 44 | 16.25977 |  | 1076.126 | 57 | 18.879 |  |
|  |  |  | 21.085873 | | | | 29.86430 | | | |
| **ΦNO** | Variance (colonies) | between | 1179.96983 | 17 | 69.40999 | **0.012** | 712.371 | 19 | 37.493 | **0.020** |
|  |  | within | 1442.05585 | 48 | 30.04283 |  | 1089.123 | 59 | 18.460 |  |
|  | Variance (tanks) | between | 425.35846 | 3 | 141.78615 | 0.011 | 439.080 | 3 | 146.360 | 0.180 |
|  |  | within | 2196.66721 | 62 | 35.43012 |  | 1362.414 | 75 | 18.166 |  |
|  | Adjusted error terms  Total phenotypic variance (*V_P_*) | | 1016.69739 | 45 | 22.59328 |  | 650.043 | 56 | 11.608 |  |
|  |  |  | 34.297454 | | | | 18.07918 | | | |
