# Supplementary material for: Estimating the Potential for Adaptation of Corals to Climate Warming
Source: PLoS One. 2010 Mar 18;5(3):e9751. doi: 10.1371/journal.pone.0009751 (PMC2841186; doi:10.1371/journal.pone.0009751)
Supplement: Table S3 — ANOVA table for symbiont pigment ratios (DT/(DD+DT) and XP/(LH+XP)). (0.05 MB DOC) [file pone.0009751.s003.doc]

**Table S3**

|  |  | | **Orpheus Isl. (*Symb*. C2)** | | | | **Magnetic Isl. (*Symb*. D)** | | | |
| --- | --- | --- | --- | --- | --- | --- | --- | --- | --- | --- |
|  |  | | **SS** | **df** | **MS** | **p** | **SS** | **df** | **MS** | **p** |
| **DT/(DD+DT)** | Variance (colonies) | between | 3.89534 E^-1^ | 17 | 2.29138 E^-2^ | **0.002** | 3.67095 E^-2^ | 19 | 1.93208 E^-3^ | **<0.001** |
|  |  | within | 3.66456 E^-1^ | 47 | 7.79693 E^-3^ |  | 1.9582 E^-2^ | 60 | 3.26366 E^-4^ |  |
|  | Variance (tanks) | between | 1.80627 E^-1^ | 3 | 6.02089 E^-2^ | 0.001 | 2.64950 E^-4^ | 3 | 8.83166 E^-5^ | 0.948 |
|  |  | within | 5.75363 E^-1^ | 61 | 9.43218 E^-3^ |  | 5.60266 E^-2^ | 76 | 7.37192 E^-4^ |  |
|  | Adjusted error terms  Total phenotypic variance (V_P_) | | 1.85829 E^-1^ | 44 | 4.22338 E^-3^ |  | 1.93171 E^-2^ | 57 | 3.38896 E^-4^ |  |
|  |  |  |  | | | | 7.37192 E^-4^ | | | |
| **XP/(LH+XP)** | Variance (colonies) | between | 4.80009 E^-3^ | 17 | 2.82358 E^-4^ | **0.003** | 2.22145 E^-3^ | 19 | 1.16918 E^-4^ | **<0.001** |
|  |  | within | 4.82567 E^-3^ | 47 | 1.02674 E^-4^ |  | 1.46449 E^-3^ | 60 | 2.44083 E^-5^ |  |
|  | Variance (tanks) | between | 2.40593 E^-4^ | 3 | 8.01977 E^-5^ | 0.669 | 4.41500 E^-5^ | 3 | 1.47166 E^-5^ | 0.820 |
|  |  | within | 9.38516 E^-3^ | 61 | 1.53855 E^-4^ |  | 3.64179 E^-3^ | 76 | 4.79184 E^-5^ |  |
|  | Adjusted error terms  Total phenotypic variance (V_P_) | | 4.58507 E^-3^ | 44 | 1.04206 E^-4^ |  | 1.42035 E^-3^ | 57 | 2.49184 E^-5^ |  |
|  |  |  | 1.487442 E^-4^ | | | | 4.79184 E^-5^ | | | |
