# Supplementary material for: Estimating the Potential for Adaptation of Corals to Climate Warming
Source: PLoS One. 2010 Mar 18;5(3):e9751. doi: 10.1371/journal.pone.0009751 (PMC2841186; doi:10.1371/journal.pone.0009751)
Supplement: Table S4 — ANOVA table for coral host gene expression (Magnetic Island). (0.05 MB DOC) [file pone.0009751.s004.doc]

**Table S4**

|  | | **Hsp70** | | | | **Ferritin** | | | |
| --- | --- | --- | --- | --- | --- | --- | --- | --- | --- |
|  |  | **SS** | **df** | **MS** | **p** | **SS** | **df** | **MS** | **p** |
| Variance (colonies) | between | 15788.236 | 19 | 830.960 | 0.087 | 22643.234 | 19 | 1191.749 | 0.486 |
|  | within | 30698.864 | 59 | 520.320 |  | 71087.295 | 59 | 1204.869 |  |
| Variance (tanks) | between | 2435.725 | 3 | 811.908 | 0.255 | 14755.480 | 3 | 4918.493 | 0.005 |
|  | within | 44051.375 | 75 | 587.352 |  | 78975.049 | 75 | 1053.001 |  |
| Adjusted error terms  Total phenotypic variance (V_P_) | | 28263.139 | 56 | 504.699 |  | 56331.815 | 56 | 1005.925 |  |
|  |  | 586.264 | | | | 1052.381 | | | |
|  | | **MnSOD** | | | | **Zn^2+^-metalloprotease** | | | |
| Variance (colonies) | between | 20947.230 | 19 | 1102.486 | **0.031** | 14079.784 | 19 | 741.041 | 0.063 |
|  | within | 34209.623 | 59 | 579.824 |  | 25734.988 | 59 | 436.186 |  |
| Variance (tanks) | between | 1170.218 | 3 | 390.073 | 0.655 | 2385.021 | 3 | 795.007 | 0.198 |
|  | within | 53986.634 | 75 | 719.822 |  | 37429.751 | 75 | 499.063 |  |
| Adjusted error terms  Total phenotypic variance (V_P_) | | 33039.405 | 56 | 589.989 |  | 23349.967 | 56 | 416.964 |  |
|  |  | 718.114 | | | | 497.983 | | | |
