# Supplementary material for: Estimating the Potential for Adaptation of Corals to Climate Warming
Source: PLoS One. 2010 Mar 18;5(3):e9751. doi: 10.1371/journal.pone.0009751 (PMC2841186; doi:10.1371/journal.pone.0009751)
Supplement: Table S5 — ANOVA table for coral host gene expression (Orpheus Island). (0.05 MB DOC) [file pone.0009751.s005.doc]

**Table S5**

|  | | **Hsp70** | | | | **Ferritin** | | | |
| --- | --- | --- | --- | --- | --- | --- | --- | --- | --- |
|  |  | **SS** | **df** | **MS** | **p** | **SS** | **df** | **MS** | **p** |
| Variance (colonies) | between | 63814.162 | 17 | 3753.774 | 0.139 | 62131.739 | 17 | 3654.808 | 0.268 |
|  | within | 118118.127 | 47 | 2513.152 |  | 137759.877 | 47 | 2931.061 |  |
| Variance (tanks) | between | 19713.785 | 3 | 6571.262 | 0.070 | 11410.100 | 3 | 3803.367 | 0.306 |
|  | within | 162218.503 | 61 | 2659.320 |  | 188481.516 | 61 | 3089.861 |  |
| Adjusted error terms  Total phenotypic variance (V_P_) | | 98404.315 | 44 | 2236.462 |  | 126349.777 | 44 | 2871.586 |  |
|  |  | 2615.790 | | | | 3067.391 | | | |
|  | | **MnSOD** | | | | **Zn^2+^-metalloprotease** | | | |
| Variance (colonies) | between | 10606 | 17 | 623.937 | 0.314 | 33683.991 | 17 | 1981.411 | **0.003** |
|  | within | 24794.385 | 47 | 527.540 |  | 33440.120 | 47 | 711.492 |  |
| Variance (tanks) | between | 3229.777 | 3 | 1076.592 | 0.117 | 4324.391 | 3 | 1541.464 | 0.222 |
|  | within | 32171.538 | 61 | 527.402 |  | 62499 | 61 | 1024.586 |  |
| Adjusted error terms  Total phenotypic variance (*V_P_*) | | 21564.608 | 44 | 490.105 |  | 28815.729 | 44 | 654.903 |  |
|  |  | 523.563 | | | | 986.530 | | | |
|  | |  | | | |  | | | |
